# Prevalence and clinical impact of severe anaemia in referral hospitals in southern Benin

**DOI:** 10.1038/s41598-025-04298-5

**Published:** 2025-07-01

**Authors:** Landry Assongba, Oumou Boukari, Jules Maroufou Alao, Natacha Nouwakpo, Jeanne Perpétue Vincent, Adrian J. F. Luty, Feiko O. ter Kuile, Achille Massougbodji, Jenny Hill, Valérie Briand, Manfred Accrombessi

**Affiliations:** 1 Institut de Recherche Clinique du Bénin (IRCB), Abomey-Calavi, Benin; 2 National Institute for Health and Medical Research (INSERM) UMR 1219, Research Institute for Sustainable Development (IRD) EMR 271, Bordeaux Population Health Centre, University of Bordeaux, Bordeaux, France; 3 Centre Hospitalier et Universitaire de la Mère et de l’Enfant Lagune (CHU-MEL), Cotonou, Benin; 4 Centre Hospitalier Départementale du Zou (CHD-Z), Abomey, Benin; 5 Université Paris Cité, IRD, MERIT, 75006 Paris, France; 6 Department of Clinical Sciences, Liverpool School of Tropical Medicine (LSTM), Pembroke Place, Liverpool, L3 5QA UK; 7 Epicentre, Médecin Sans Frontière, Paris, France

**Keywords:** Prevalence, Cross sectional survey, Severe anaemia, In-hospital mortality, Children, Benin, Epidemiology, Paediatric research, Infectious diseases

## Abstract

Severe anaemia is a critical public health issue worldwide, disproportionately affecting children in Africa, where approximately 40% of children aged 6 to 59 months are impacted. It leads to significant hospital and post-hospital complications. However, there is a notable lack of research on its burden and clinical impact, particularly in Benin, where existing data are outdated. This study aims to assess the prevalence and impact of severe anaemia in two areas of perennial transmission in southern Benin. We conducted a retrospective cross-sectional study at two referral hospitals in Benin, Lagune Mother and Child University Hospital Centre (CHU-MEL) and the Departmental Hospital Centre-Zou (CHD-Z). Sociodemographic, clinical and biological information were extracted from medical records of patients admitted to paediatric ward in 2023, using a standardised questionnaire. Clinical severe anaemia was defined as anaemia with decompensation requiring blood transfusion, and biological severe anaemia as haemoglobin < 5 g/dl. A total of 7152 paediatric hospital records were included in the analysis (CHU-MEL = 4388; CHD-Z = 2764). The median (IQR, range) age was 32 (13–61; 1–228) months. Severe malaria (N = 3653/7152 [51.1%]) and clinical severe anaemia (3586/7152 [50.1%]) were the most common diagnoses with four out of five children (2836/3586 [79.0%]) clinically diagnosed with severe anaemia cases had malaria. In children with severe anaemia, the risk of death decreased slightly with year of age (aOR 0.95, 95% CI 0.92–0.99, *p* = 0.019). In contrast, severe malnutrition increased the risk of death (aOR 1.80, 95% CI 1.33–2.43, *p* < 0.001) being significant risk factors. Severe anaemia is a major contributor to paediatric hospital admissions, with severe malaria being a leading cause in these regions. This study highlights the critical need for a comprehensive management strategy for severe anaemia, particularly in the youngest children. An integrated strategy of effective malaria chemoprevention, such as post-discharge malaria chemotherapy combined with targeted nutritional interventions, are essential to mitigate mortality rates in areas with high malaria transmission, where the dual burden of malaria and nutritional deficiencies exacerbates paediatric morbidity and mortality.

**Supplementary Information:**

The online version contains supplementary material available at 10.1038/s41598-025-04298-5.

## Introduction

Severe anaemia remains a critical global public health concern, disproportionately affecting children in Africa. It is estimated that 40% of children aged 6 to 59 months worldwide suffer from anaemia, and this age group accounts for a substantial proportion of anaemia-related mortality. Africa and South-East Asia are the most affected regions, together contributing to approximately 40% of anaemia cases among children under five^1,2^. It is particularly prevalent in low- and middle-income countries, where children under five experience disproportionately high mortality rates^3^. In sub-Saharan Africa, severe anaemia is a leading cause of hospitalisation and death, largely driven by factors such as nutritional iron deficiency, soil-transmitted helminths, and malaria^2,4^. It is a multifactorial condition, often associated in African countries to specific diseases such as malaria, hookworm and HIV, or with individual factors like nutritional deficiencies in vitamin A or vitamin B12^5^. Despite its widespread prevalence and severe consequences, anaemia remains under-researched. Existing studies suggest that anaemia is associated with significant “hidden” morbidity and mortality, often manifesting months after diagnosis and treatment^3^.

In malaria-endemic regions, anaemia and malaria often coexist, with the prevalence of malaria infection strongly correlated with anaemia rates^6^. Among children under 15 years of age, anaemia prevalence generally increases with parasite density. However, this relationship can be bidirectional: a high parasite density can lead to anaemia, and conversely, anaemia can predispose to more serious parasite infections by impairing immune function^6^. Data on severe anaemia remain limited, and existing data generally come from disease-focused studies or sub-populations such as hospitalised patients^7–9^. In Benin, severe anaemia related to malaria is particularly prevalent in paediatric hospitals^10^. For instance, in northern Benin, 52.6% of severe malaria admissions had severe anaemia, hypoglycaemia, or both^11^. According to the last Demographic and Health Survey conducted in Benin in 2018, the prevalence of severe anaemia in children under five years of age was estimated at 3.3%^12^. Despite its importance, few studies have comprehensively assessed the broader scope of severe anaemia in Benin, including its underlying causes and its impact on both hospital outcomes and post-discharge recovery. While previous studies in Beninin 2015 and 2018 have examined severe anaemia specifically in malnourished children^13,14^, or in the context of transfusion practices in 2024^15^, our study provides a broader perspective by investigating severe anaemia in referral hospitals, and assessing its prevalence and clinical impact.

There is a critical need to assess the burden of severe anaemia in Benin, and its clinical impact on hospital outcomes. The present study aims to quantify the burden of severe anaemia, its associated co-morbidities, and its clinical impact on in-hospital mortality in two geographically distinct referral hospitals in southern Benin.

## Methods

### Study context

This study was part of the pre-intervention phase to collect baseline data for a cluster-randomised implementation trial designed to assess different strategies for optimising health system delivery of and adherence to post discharge malaria chemotherapy (PDMC) in Benin, an integral part of the EDCTP-funded “PDMC Saves Lives” project (ClinicalTrial.gov, NCT06601712: September 2024; PACTR202411682724094). PDMC is recommended by the World Health Organisation (WHO) for the post-discharge management of children with severe anaemia in settings with moderate to high perennial malaria transmission to reduce hospital readmissions and deaths after discharge.^16^.

### Study design

We conducted a retrospective cross-sectional survey in two referral hospitals in Benin: one located in the Littoral region (Lagune Mother and Child University Hospital Centre, CHU-MEL) and the other in the Zou region (Departmental Hospital Centre-Zou (CHD-Z)). The CHU-MEL hospital is a national referral centre focused on mother and child health, situated in Cotonou, the economic capital of Benin. The CHD-Z is the regional referral hospital serving the Zou and Collines areas and is located in Abomey, approximately 150 km from Cotonou. These hospitals were selected as part of the PDMC-SL trial not only because of their status as referral hospitals with adequate technical facilities for severe anaemia management, but also because of their capacity to attract a high annual volume of children from various localities within their catchment areas.

Data collection occurred from March 25, 2024, to May 13, 2024 and encompassed all paediatric admissions to the general paediatric wards of both hospitals during the year 2023. General paediatrics serve children aged 1 month to 18 years. Data were extracted from the medical records of these children using a standardised data collection questionnaire. Neonatal admissions were excluded from the analysis.

### Study procedures

Information collected included the child’s age, sex, suspected diagnosis at admission, and confirmed clinical diagnoses as documented by the health care providers. For children diagnosed with severe malaria and/or severe anaemia at admission or during hospitalisation, additional data were gathered. These included anthropometric measurements, vaccination status, HIV status, admission and discharge dates, mode of admission, and any history of severe anaemia within the past year. Details of treatments were also recorded, covering details on parenteral therapies administered during hospitalisation, oral antimalarial treatment prescribed at discharge, and clinical outcomes of disease.

Furthermore, we documented the geographical residence (department, commune, arrondissement, and village) of children admitted for severe anaemia. Finally, when available, we recorded the results of relevant biological tests, including Hb levels, thick blood smear, urine culture, C-reactive protein, blood culture results.

### Definition of variables

(i) Clinical severe anaemia: this refers to a clinical diagnosis of severe anaemia as documented in the medical records. Although the definition may vary slightly based on clinical judgment, it generally includes the presence of palmar pallor, coupled with signs of decompensation (cold extremities, altered consciousness, lower limb oedema, respiratory distress, chest indrawing, intercostal retractions, nasal flaring, increased jugular venous pressure, cardiac failure, or shock).
(ii) Severe biological anaemia: defined as a haemoglobin level < 5 g/dl^17,18^.
(iii) severe malarial anaemia: was defined as severe anaemia in the presence of any evidence of malaria infection detected by rapid diagnostic tests or microscopy^19^.
(iv) Nutritional status: was assessed using weight-for-height (WHZ), height-for-age (HAZ), and weight-for-age (WAZ) z-scores. Severe malnutrition was defined as at least one z-score < − 3, moderate malnutrition as at least one z-score between − 3 and − 2, and no malnutrition as z-scores ≥ − 2^20^.
(v) Comorbidity: defined as the presence of two or more concurrent diseases or medical conditions in the same patient. In addition to severe anaemia, medical records frequently documented other conditions, including severe malaria, malnutrition, hemoglobinopathies, gastrointestinal diseases, cardio-respiratory diseases and infectious syndromes.
(vi) In-hospital mortality: defined as any death occurring during hospitalisation among children admitted to one of the two study hospitals in 2023.
(vii) Clinical outcomes: defined as the final outcome of a patient’s hospitalisation, reflecting the patient’s state of health at the end of their hospital stay. In this study, clinical outcomes are categorised as follows: death during hospitalisation, clinically cured and discharged, discharged against medical advice or referred to another institution for follow-up or specialist care.

### Data management and statistical analysis

Data were collected using a standardised questionnaire and entered using KoboCollect v2024.1.3 (developed by the Harvard Humanitarian Initiative), an application installed on tablets and smartphones. Data were transmitted daily to a secure, encrypted server. All data extractions were performed in CSV format, and subsequent analyses were conducted using Stata 15.0 (STATA Corporation, College Station, Texas). No information that would enable patients to be identified directly was collected, and a numeric code was assigned to ensure anonymity and confidentiality throughout the data collection and analysis process.

Data analysis was stratified by study site. The overall prevalence of clinical severe anaemia was calculated as the total number of severe anaemia cases divided by the total inpatient population, with 95% confidence intervals (CI) provided. It was used as a severe anemia variable for the study’s various descriptive and comparative analyses, since it also includes biological severe anaemia. Monthly prevalence rates were also computed to assess potential temporal variations. Descriptive analyses were performed to summarise patient characteristics, including age, gender, origin, HIV status, nutritional status and other comorbidities. Data were presented using means or medians for continuous variables and proportions for categorical variables. The Pearson Chi^2^ or Fisher exact tests were used for comparisons between groups for categorical variables. The Mann–Whitney test was used for comparison of age distributions by in-hospital death status. A bivariate analysis was performed to explore the association between in-hospital mortality related to severe anaemia and potential risk factors (age, sex, child nutritional status, child immunisation status, hospital visited and severe malarial anaemia). A backward stepwise procedure was used to select variables associated with deaths among children admitted to hospital with severe anaemia. A multivariate logistic regression model included variables associated with in-hospital mortality at a significance level of 0.2 in the bivariate analysis.

### Ethical considerations

The study protocol was approved by the Ethics and Research Committee of the Institute of Applied Biomedical Sciences (CER-ISBA, N°191 of 18/12/2023). The study was conducted in compliance with the ethical principles from the revised Declaration of Helsinki, guaranteeing the protection of the rights, dignity and confidentiality of the participants.

In accordance with Article 23 of Law No. 2010-40 of 08 December 2010 on the Code of Ethics and Professional Conduct for Health Research in the Republic of Benin, informed consent was not required for this retrospective study based on hospital medical records. No information that could identify the participants was collected, and no contact with the participants was made during data collection. In addition, the study was conducted in referral hospitals with university affiliation, and site-specific research authorisations were obtained.

## Results

The final sample consisted of 7152 records, with 4388 from CHU-MEL and 2764 from CHD-Z. The median age (IQR); Range was 32 months (13–61); 1–228 with a slight predominance of males (3853/7152 [53.9%]) (Table 1). Severe malaria and clinical severe anaemia were the leading causes of hospitalisation, accounting for (3653/7152 [51.1%]) and (3586/7152 [50.1%]) of admissions respectively. Less common diagnoses at admission included malnutrition, cardio-respiratory conditions, gastrointestinal diseases, and sickle cell anaemia (appendix).

**Table 1:** Socio-demographic and clinical characteristics of the study population.

Characteristics		All	CHU-MEL	CHD-Z
Median age in months (IQR; Range)		32 (13–61; 1–228)	36 (14–60; 1–207)	30 (12–72; 1–228)
Mean age in months (standard deviation)		47.1 (± 45.46)	46.2 (± 43.18)	48.5 (± 48.84)
Proportion of male children (%; n/N)		53.9; 3853/7152	53.1; 2330/4388	55.1; 1523/2764
Proportion of children with severe malaria (%; n/N)		51.1; 3653/7152	62.0; 2720/4388	33.8; 933/2764
Severe malaria parenteral treatment (%; n/N)	Artesunate injectable	92.3; 3370/3653	97.6; 654/2720	76.7; 716/933
	Artemether injectable	0.03; 1/3653	0	0.1; 1/933
Children tested for malaria (%; n/N)		47.7; 3409/7152	57.9; 2539/4388	31.5; 870/2764
Malaria test result (%; n/N)	Negative	19.6; 668/3409	18.2; 463/2539	23.3; 205/870
	Positive	80.4; 2741/3409	81.8; 2076/2539	76.4; 665/870
Proportion of children with severe clinical anaemia (%; n/N)		50.1; 3586/7152	56.0; 2453/4388	41.0; 1133/2464
Children tested for Hb among clinical severe anaemia (%; n/N)		92.1; 3302/3586	93.2; 2285/2453	89.8; 1017/1133
Hb level (g/dL) (%; n/N)	≥ 11	0.7; 22/3302	0.5; 11/2285	1.1; 11/1017
	5–10.9	50.0; 1652/3302	49.0; 1120/2285	52.3; 532/1017
	< 5	49.3; 1628/3302	50.5; 1154/2285	46.6; 474/1017
Severe malarial anaemia (%; n/N)		79.0; 2834/3586	88.7; 2175/2453	58.2; 659/1133
Proportion of children died while in hospital (%; n/N)		10.5; 376/3586	9.1; 223/2453	13.5; 153/1133
Children discharged among severe clinical anemias (%; n/N)		80.5; 2886/3586	85.5; 2098/2453	69.5; 788/1133
Children treated with an oral antimalarial at hospital discharge (%; n/N)		85.9; 2479/2886	92.6; 1942/2098	68.2; 537/788
Received antimalarial drug type (%; n/N)	None	14.1; 407/2886	7.4; 156/2098	31.9; 251/788
	Artemether-lumefantrine cp	37.9; 1093/2886	26.7; 560/2098	67.6; 533/788
	Dihydroartemisinin-piperaquine cp	47.8; 1380/2886	65.6; 1377/2098	0.4; 3/788
	Other (Quinine, ASAQ, SP)	0.2; 6/2886	0.2; 5/2098	0.1; 1/788
Proportion of children transfused (%; n/N)		47.1; 3365/7152	53.5; 2347/4388	36.8; 1018/2764
Proportion of severe anaemia transfused (%; n/N)		88.2%; 3164/3586	88.7; 2175/2453	87.3; 989/1133
Average haemoglobin levels in children transfused (standard deviation)		4.98 (± 1.66)	4.91 (± 1.54)	5.15 (± 1.88)
Median hospital stays in days (IQR)		4 (2–6)	3 (2–5)	5 (3–7)
Child’s vaccine status among severe clinical anemias (%; n/N)	Up-to-date	43.5; 1561/3586	49.5; 1214/2453	30.6; 347/1133
	Not up-to-date	17.5; 628/3586	15.7; 384/2453	21.5; 244/1133
	Don’t know	36.4; 1306/3586	33.4; 820/2453	42.9; 486/1133
HIV test among severe clinical anemias (%; n/N)		50.3; 1803/3586	34.9; 856/2453	83.6; 947/1133
HIV-seropositive children (%; n/N)		0.9; 44/1803	0.7; 26/856	1.5; 18/947
Mode of admission (%; n/N)	Caregivers	28.4; 1017/3586	26.1; 640/2453	26.1; 377/1133
	Referral from a hospital	63.8; 2286/3586	66.6; 1633/2453	57.6; 653/1133

The proportions of malaria test results are calculated only for children admitted with severe malaria who were tested.

The proportions of haemoglobin levels are calculated only for children diagnosed with severe anaemia who had an Hb.

Severe malaria anaemia is defined as the proportion of cases of severe anaemia who received parenteral antimalarial treatment in hospital during this episode.

**Fig. 1:**
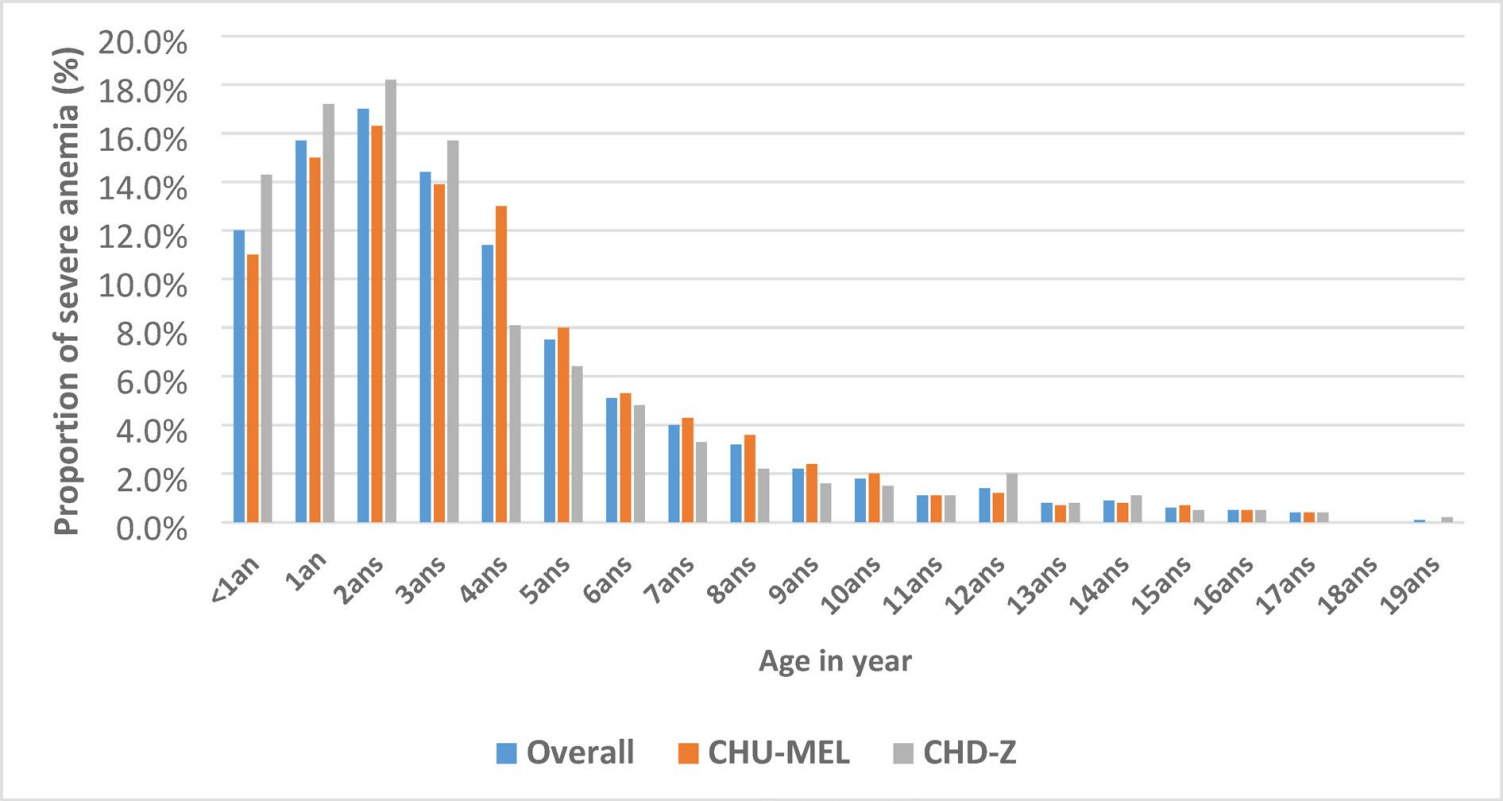
Distribution of severe anaemia in the two referral paediatrics hospitals according to age.

Nearly all severe malaria cases were treated with injectable artesunate (3370/3653 [92.3%]). Malaria diagnostic confirmation, using either rapid diagnostic tests or thick blood smear, was positive in 80.4% of cases (2741/3409). The prevalence of clinical severe anaemia was 50.1% (3586/7152). Among these children, 49.3% (1628/3302) had haemoglobin levels < 5 g/dL (biological severe anaemia), with similar estimates across both hospitals (Table 1). Severe anemia prevalence was highest at the age of 2 years and remains above 10% for each groupe up to 4 years of age; and then decreases significantly after five years, it and drops below 5% (Fig. 1).

Severe malaria was identified as the primary cause of severe anaemia (79.0%). This proportion reached 88.7% (2175/2453) at CHU-MEL and 58.2% (659/1133) at CHD-Z. Most of the children recovered after treatment; however, 10.5% (376/3586) of those hospitalized with severe anaemia died, including 9.1% at CHU-MEL and 13.5% at CHD-Z. Among survivors, 92.6% (1942/2098) at CHU-MEL and 68.2% (537/788) at CHD-Z were prescribed antimalarial drugs upon hospital discharge. Dihydroartemisinin-piperaquine (DHA-PP) was the most commonly prescribed oral antimalarial at CHU-MEL (1377/2098 [65.6%]), whereas artemether-lumefantrine (AL) was the preferred regimen at CHD-Z (533/788 [67.6%]) (Table 1). Additionally, nearly half (3365/7152 [47.1%]), of the hospitalized children received a blood transfusion due to their clinical condition. The mean haemoglobin level among hospitalised children were 4.98 g/dl (± 1.66). (Table 1).

**Fig. 2:**
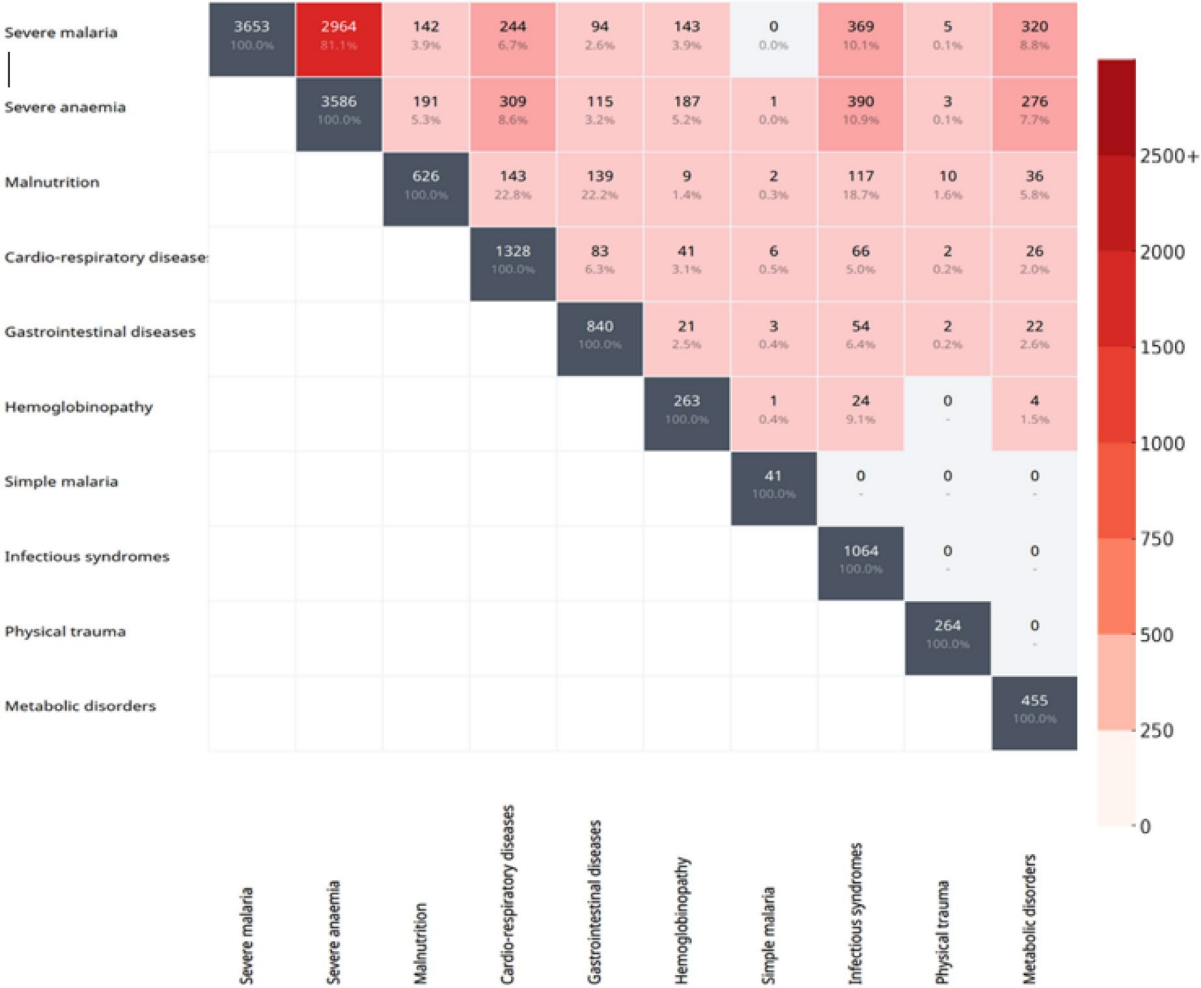
Co-occurrence matrix for diagnoses of children admitted to paediatric hospitals in 2023.

The median duration of hospitalisation was 4 days (Interquartile range [IQR]: 2–6) across both hospitals, with CHU-MEL showing a shorter median duration of 3 days (IQR: 2–5) compared to CHD-Z, where the median duration was 5 days (IQR: 3–7). Approximately 17.5% (638/3586) of children with clinical severe anaemia had incomplete vaccination records. HIV positivity was rare, with less than 1% of children testing positive. Additionally, 63.8% (2286/3586) of the children were referred from peripheral healthcare centres (Table 1). A significant number of hospitalised children (2964 out of 7152) had both severe anaemia and severe malaria. (Fig. 2).

**Fig. 3:**
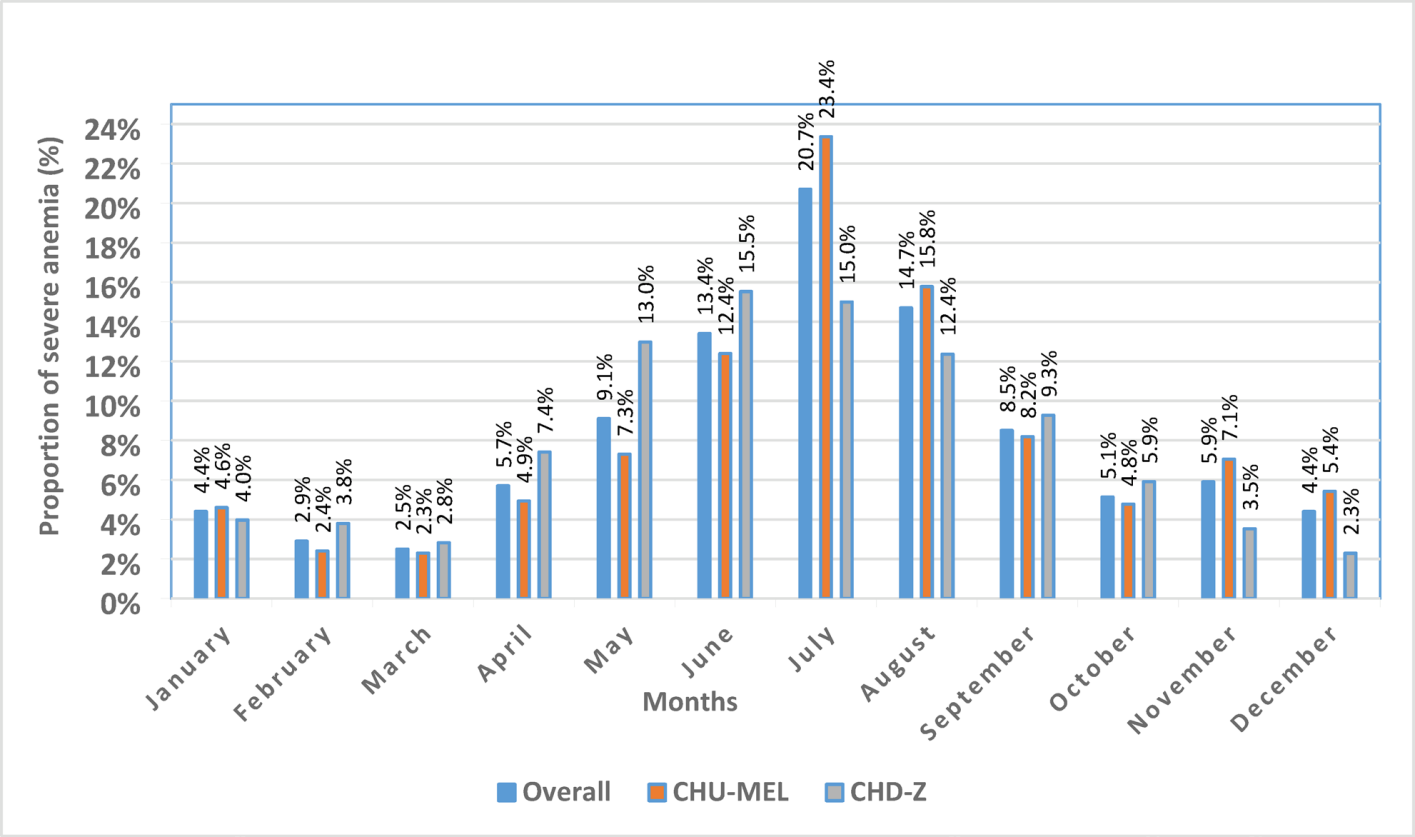
Temporal variation in severe clinical anaemia in paediatrics hospitals in 2023.

Severe anaemia cases showed a seasonal pattern, with the highest cases occurring from May to September 2023. The peak incidence was observed in June-July, accounting for 20.7% of all severe anaemia cases at CHU-MEL. This trend was consistent across both sites (Fig. 3). Regarding spatial distribution, CHU-MEL received the highest number of severe anaemia cases from the communes of Abomey-Calavi (41.3%) and Cotonou (17.9%). In Abomey-Calavi, the Godomey (29.4%) and Hêvié (28.5%) arrondissements were the primary sources, while in Cotonou, the 12th arrondissement (17.9%) contributed the majority of cases. At CHD-Z, the commune of Bohicon was the most affected (27.8%), with the Bohicon I (33%) and Bohicon II (25.1%) arrondissements accounting for most severe anaemia admissions (appendix 2).

**Fig. 4:**
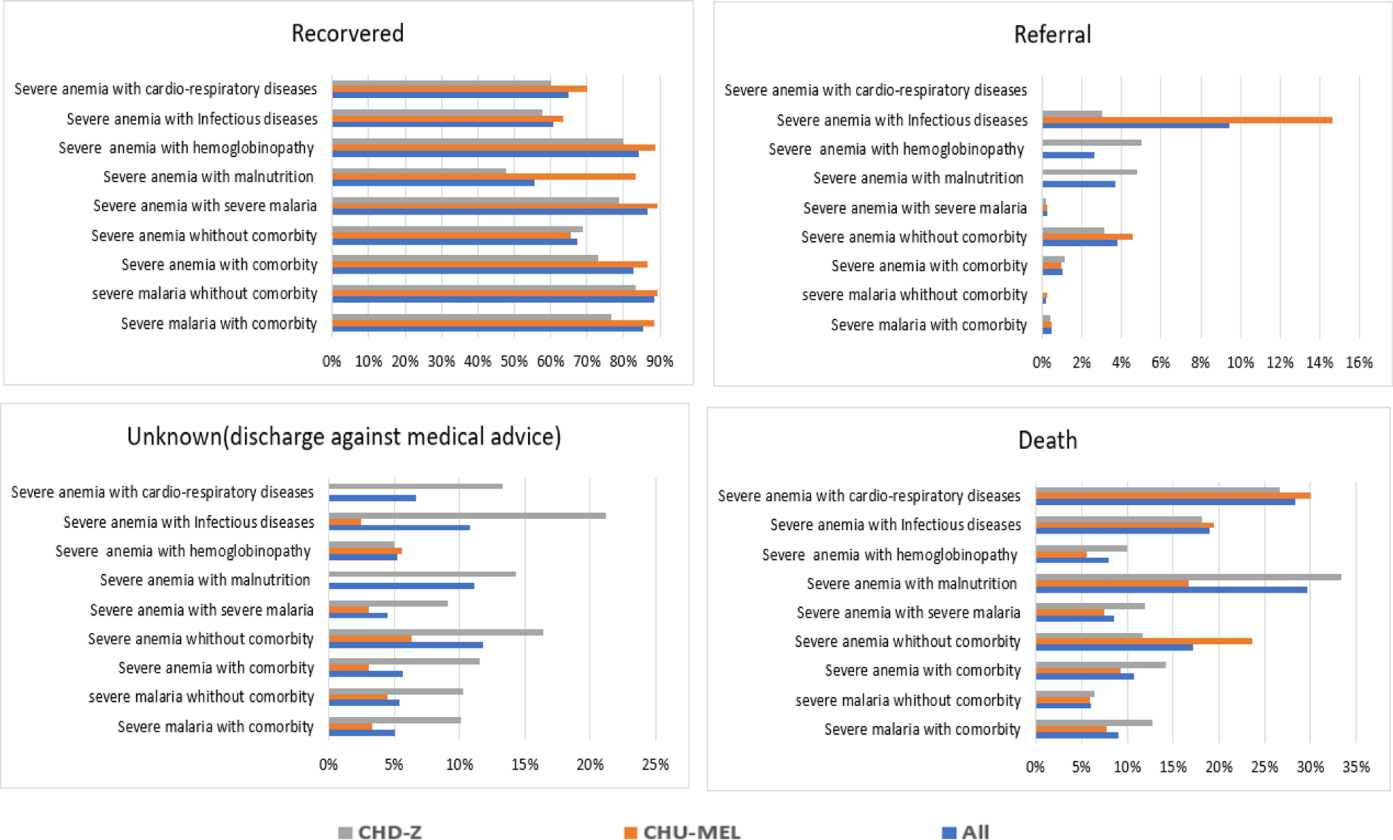
Clinical outcome of children according to priority diagnoses.

About one-in-ten children with severe anaemia (10.5%, 376/3586) died. Overall, mortality was particularly high when severe anaemia coexisted with malnutrition (29.3%) or cardiopulmonary diseases (28.3%). At CHU-MEL, mortality rates were higher when severe anaemia coexisted with comorbid conditions such as cardio-respiratory pathologies (30%), malnutrition (16.7%), or infectious syndromes (19.5%). Mortality was also notably high when severe anaemia occurred without any other associated morbidities (23.6%). At CHD-Z, mortality rate was also higher when severe anaemia coexisted with malnutrition (33.3%) or cardio-respiratory pathologies (26.7%). (Fig. 4).

In-hospital mortality related to severe anaemia was significantly associated with age, nutritional status, hospital visited and the presence of severe malarial anaemia. After adjusting for confounding factors, each additional year of age was associated with a 5% reduction in the risk of death (adjusted odds ratio [aOR] 0.95, 95% CI 0.92–0.99, *p* = 0.019). Children with severe malnutrition were 80% more likely to die (adjusted odds ratio [aOR] 1.80, 95% CI 1.34–2.43, *p* < 0.001). In addition, children without severe malaria-related anaemia were 4.70 times more likely to die than those with severe malaria-related anaemia (adjusted odds ratio [aOR] 4.70, 95% CI 3.74–5.89, *p* < 0.001) (Table 2).

**Table 2:** Factors associated with death in children hospitalised for severe anaemia.

Clinical characteristics	In-hospital death							
	Descriptives		Bi-variate analysis			Multivariate analysis		
	n/N	%	OR	95%CI	*P*-value	aOR	95%CI	*P*-value
Age (year)	376/3495	10.8	0.93	0.89–0.96	** < 0.001**	0.95	0.92–0.99	0.019
Nutritional status					** < 0.001**			
No malnutrition	208/2332	8.9	1	–	–	1	–	–
Moderate malnutrition	80/666	12.0	1.39	1.06–1.83	0.017	1.28	0.95–1.72	0.100
Severe malnutrition	88/497	17.7	2.19	1.67–2.88	< 0.001	1.80	1.33–2.43	< 0.001
Severe malarial anaemia					** < 0.001**			
Yes	200/2834	26.6	1	–	–	1	–	–
No	176/661	7.1	4.77	3.82–5.98	< 0.001	4.70	3.74–5.89	< 0.001
Referral hospital								
CHU-MEL	223/2418	9.2	1	–	–			
CHD-Z	153/1077	14.2	1.63	1.31–2.03	< 0.001			

Nutritional status: Assessed using WHZ, HAZ, and WAZ z-scores. Severe malnutrition: any z-score < − 3; moderate malnutrition: any z-score between − 3 and − 2; no malnutrition: all z-scores ≥ − 2. according to WHO standards.

Significant values are in [bold].

## Discussion

This study aimed to assess the prevalence and outcomes of severe anemia in children hospitalized in two referral hospitals in southern Benin. The burden of severe anemia was significant, with 4 out of 5 cases due to severe malaria. The hospital mortality rate was higher when severe anemia coexisted with malnutrition (29.3%). Additionally, age, the hospital visited, and malnutrition were strongly associated with an increased risk of hospital mortality.

Severe anemia was predominant among hospital admissions, accounting for half of the cases. This high hospital prevalence reflects the fact that hospitalized children often present more severe clinical conditions, such as severe malaria, malnutrition or bacterial infections, and are therefore different from the general paediatric population. Even so, this rate aligns with findings from other studies conducted in West and Central Africa^14,21^. In contrast, lower proportions of anemia have been reported in East Africa (e.g., Kenya and Tanzania) and North Africa (e.g., Sudan)^8,22,23^. Most of these studies, except for the one in Sudan, focused on children under 5 years old. The proportion of severe anemia peaked at two years of age, remained above 10% until four years, and then significantly declined after five years. In this study, 79% of severe anemia cases were diagnosed with severe malaria. In comparison, a keynian study conducted in 2007 reported a higher proportion, with 85% of severe anemia cases linked to severe malaria^8^. Beyond malaria, nutritional deficiencies, particularly iron deficiency, are significant contributors to severe anemia. Yessoufou et al*.* found that iron deficiency was responsible for 78.8% of severe anemia cases. Poor weaning practices and inadequate consumption of iron-rich foods further exacerbated the risk of nutritional anemia in this population^14^. However, the aetiology of severe anaemia in children is often multifactorial. In addition to nutritional deficiencies, other contributors include chronic inflammatory conditions, parasitic worm infestations (such as hookworm or schistosomiasis), and repeated infections, all of which can impair red blood cell production or increase destruction^24^. In severe malaria, high parasitemia combined with a strong inflammatory response can rapidly lead to severe haemolysis and bone marrow suppression, contributing to acute drops in haemoglobin levels^25^. Nearly half of the hospitalized children had been transfused (47.1%), reflecting the critical severity of anemia cases. This observation is slightly lower but consistent with previous studies conducted in Burkina Faso and Benin (CHU-MEL), which reported transfusion rates of 78% and 63%, respectively^26,27^.

The median hospital stay was four days, longer at CHD-Z compared to CHU-MEL. These prolonged stays are likely due to the need for comprehensive management, addressing both the immediate treatment of anemia and its underlying causes^22,28^. Mortality rates were higher when severe anemia was associated with comorbidities, such as cardio-respiratory diseases, malnutrition, or infectious syndromes. Notably, malnutrition was particularly associated with poor outcomes, as one-third of children with both severe anemia and malnutrition died at CHD-Z. Furthermore, hospital mortality related to severe anemia was strongly influenced by age, nutritional status and the presence of malaria-related anemia. Older children had a lower risk of death, while those suffering from malnutrition had a higher risk, particularly the most severely malnourished. Finally, children without malaria-related anemia had a significantly higher risk of death. This phenomenon may be explained by the fact that severe malaria-related anemia is generally identified quickly and treated according to well-established protocols, thereby reducing the risk of mortality. Conversely, when the underlying cause of anemia is not promptly identified or when hospital admission is delayed, the administration of appropriate care may be postponed, increasing the risk of death.

Severe malaria emerged as a co-principal cause of hospitalization, accounting for more than half of pediatric admissions, along with severe anemia. This finding aligns with studies conducted in Burkina Faso, where severe malaria represented 46% of pediatric admissions^26^, and in Kenya, where 83% of children under five were reported to have malaria parasitemia^8^. In Benin, severe malaria remains a major cause of hospitalization among children under five, as reported in similar studies^14,27^. The variability in malaria prevalence across regions can be attributed to several factors, including the implementation of malaria control measures, local vector resistance, differences in healthcare facilities, and geographical variations^29^.

Seasonal variations in severe anemia cases were observed, with peaks between May and September, particularly in June and July, corresponding to the rainy season. This period coincides with a well-documented increase in malaria cases in endemic regions^28^. In this context, the PDMC-SL study, based on the WHO’s new recommendation for post-hospitalization malaria chemoprevention in children treated for severe anemia, holds great potential, especially in malaria-endemic areas. This approach aims to prevent malaria recurrence and its associated complications in vulnerable populations. However, its efficacy and feasibility need to be thoroughly evaluated before broader adoption, including determining the optimal drug regimen and implementation strategies. The PDMC-SL implementation trial will be essential in addressing these gaps, generating context-specific data to inform its application in Benin^30^.

This study has certain limitations that may affect the interpretation of its results. Firstly, the lack of precise data on the delay between symptom onset and hospitalization limits the understanding of healthcare-seeking behaviors influencing severe anemia outcomes. The hospital-based study design may not accurately represent the prevalence of severe anemia among children who do not seek care or are treated in other healthcare facilities. Additionally, the focus on data from two referral hospitals, identified in the PDMC-SL study, limits its representativeness. Furthermore, retrospective data extracted from medical records may be affected by information quality, including incomplete documentation or transcription errors. Despite these constraints, the study provides crucial epidemiological insights into severe anemia in southern Benin, contributing to future control and optimization efforts within the PDMC-SL framework. It also highlights the need to adopt an integrated approach, including the treatment of underlying causes of anaemia, particularly in young children, and other key factors such as the promotion of good nutrition.

## Conclusion

Severe anaemia remains a major cause of paediatric hospital admissions in southern Benin, with mortality influenced by younger age, malnutrition and the underlying cause of anaemia. Each additional year of age is associated with a reduced risk of death, highlighting the increased vulnerability of the youngest children. Malnutrition also plays a key role, with severely malnourished children running a significantly higher risk of death. In addition, children with severe non-malarial anaemia have an increased risk of death, probably due to delays in diagnosis and treatment, whereas well-established protocols for malaria-related anaemia may have a protective effect. These findings underscore the need for an integrated approach that combines prompt malaria diagnosis and treatment with robust nutritional support. Strengthening early detection of non-malarial causes of anaemia and improving overall care strategies by management of underlying causes of anemia, promotion good nutrition, worm control and multivitamin supplementation are essential to reduce mortality. Further research is needed to improve our understanding of the epidemiology of severe anaemia and to inform the development and optimisation of targeted public health strategies within frameworks such as the PDMC-SL to improve prevention and treatment outcomes for vulnerable paediatric populations.

## Electronic supplementary material

Below is the link to the electronic supplementary material.

Supplementary Material 1

## Data Availability

The datasets are available from the corresponding authors on a reasonable request.

## References

[1] Organisation Mondiale de la Santé (OMS). Anémie [Internet]. 2023 [cité 7 oct 2024]. Disponible sur: https://www.who.int/fr/news-room/fact-sheets/detail/anaemia

[2] Apouey, B., Picone, G., Wilde, J., Coleman, J. & Kibler, R. Malaria and anemia among children in sub-Saharan Africa: The effect of mosquito net distribution. *Revue Économique* **68**(2), 163–197 (2017).

[3] Phiri, K. S. et al. Long term outcome of severe anaemia in Malawian children. *PLoS ONE* **3**(8), e2903 (2008). 18682797 10.1371/journal.pone.0002903 PMC2488370

[4] Global Health Metrics. Institute for Health Metrics and Evaluation. [cité 7 oct 2024]. Anémie—Déficience de niveau 1|Institut de mesure et d’évaluation de la santé. Disponible sur: https://www.healthdata.org/research-analysis/diseases-injuries-risks/factsheets/2021-anemia-level-1-impairment

[5] van Hensbroek, M. B., Jonker, F. & Bates, I. Severe acquired anaemia in Africa: new concepts. *Br. J. Haematol.* **154**(6), 690–695. 10.1111/j.1365-2141.2011.08761.x (2011). 21707575 10.1111/j.1365-2141.2011.08761.x

[6] Ouedraogo, O. et al. Anémie et infection palustre chez des enfants de moins de 15 ans vivant en zone endémique du paludisme au Burkina Faso. *Int. J. Biol. Chem. Sci.* **17**(7), 2653–2662 (2023).

[7] Malamba, S. et al. The effect of HIV on morbidity and mortality in children with severe malarial anaemia. *Malar J.* **6**, 143 (2007). 17973997 10.1186/1475-2875-6-143 PMC2170443

[8] Obonyo, C. O., Vulule, J., Akhwale, W. S. & Grobbee, D. E. In-hospital morbidity and mortality due to severe malarial anemia in western Kenya. *Am. J. Trop. Med. Hygiene* **77**(6), 23–28 (2007). 18165471

[9] Bouyou-Akotet, M. K. et al. Impact of *Plasmodium falciparum* infection on the frequency of moderate to severe anaemia in children below 10 years of age in Gabon. *Malar J.* **8**, 166 (2009). 19619296 10.1186/1475-2875-8-166 PMC2722664

[10] JEAI TILAPIA : Paludisme grave: formes cliniques ; étiologies des Anémies et des sepsis associés, en pédiatrie. | Site Web IRD. [cité 7 oct 2024]. Disponible sur: https://www.ird.fr/jeai-tilapia-paludisme-grave-formes-cliniques-etiologies-des-anemies-et-des-sepsis-associes-en

[11] Adedemy, J. D., Agossou, J., Alao, M. J., Noudamadjo, A. & Ayivi, B. Role of severe anemia and hypoglycemia in severe malaria’s mortality in children in a pediatric ward in Parakou (Benin). *Mali Med.* **30**(1), 19–24 (2015). 29927153

[12] Institut National de la Statistique et de l’Analyse Économique (INSAE) et ICF. Enquête Démographique et de Santé au Bénin, 2017–2018. Cotonou, Bénin et Rockville, Maryland, USA: INSAE et ICF. [cité 7 oct 2024]. Disponible sur: https://instad.bj/images/docs/insae-statistiques/enquetes-recensements/EDS/2017-2018/1.Benin_EDSBV_Rapport_final.pdf (2019).

[13] Adebo, A. A., Yessoufou, A. G., Behanzin, J. G., Kabanoude, A. A. & Yessoufou, A. K. Anémie chez les enfants de moins de 5 ans reçus en consultation au service de pédiatrie de l’Hôpital de Zone d’Abomey-Calavi/So-Ava (Sud du Bénin). *J. Appl. Biosci.* **123**, 12373–12378 (2018).

[14] Yessoufou, A. G. et al. Prévalence de l’anémie chez les enfants malnutris de 6 à 59 mois hospitalisés au CHD-Zou dans le plateau d’Abomey (Centre du Bénin). *Int. J. Biol. Chem. Sci.* **9**(1), 82–90 (2015).

[15] Agbeille, M. F. et al. Transfusion sanguine dans le service de pédiatrie du Centre Hospitalier Universitaire du Borgou/Alibori au Bénin. *Journal de la Recherche Scientifique de l’Université de Lomé.* **26**(1), 221–230 (2024).

[16] World Health Organisation Global Malaria Programme. WHO guidelines for malaria. Disponible sur: https://iris.who.int/handle/10665/354781 (2022).

[17] Severe Malaria Observatory. Knowledge sharing for severe malaria. Severe Malaria Criteria, Features & Definition. Disponible sur: https://www.severemalaria.org/severe-malaria/severe-malaria-criteria-features-definition

[18] World Health Organization (WHO). Guidelines for the treatment of malaria. Third edition|WHO |Regional Office for Africa. Disponible sur : https://www.afro.who.int/publications/guidelines-treatment-malaria-third-edition (2024).

[19] Kwambai, T. K. et al. Malaria chemoprevention in the postdischarge management of severe anemia. *N. Engl. J. Med.* **383**(23), 2242–2254 (2020). 33264546 10.1056/NEJMoa2002820 PMC9115866

[20] World Health Organization (WHO). Malnutrition. Disponible sur: https://www.who.int/news-room/fact-sheets/detail/malnutrition (2024).

[21] Mutombo, A. M. et al. Severe malaria and death risk factors among children under 5 years at Jason Sendwe Hospital in Democratic Republic of Congo. *Pan. Afr. Med. J.* **29**, 184 (2018). 30061962 10.11604/pamj.2018.29.184.15235 PMC6061819

[22] Msinde, P. S. The prevalence, etiology, and outcome of anemia in children under five on admission in three hospitals of dar-es-salaam. *medRxiv*10.1101/2023.12.29.23300509v1 (2023).

[23] Ahmed, M. A. A., Al-Nafeesah, A., Al-Wutayd, O., Mahgoub, H. M. & Adam, I. Severe childhood anemia and emergency blood transfusion in Gadarif Hospital, eastern Sudan. *PLoS ONE* **14**(12), e0225731. 10.1371/journal.pone.0225731 (2019). 31794569 10.1371/journal.pone.0225731 PMC6890167

[24] Kassebaum, N. J. et al. A systematic analysis of global anemia burden from 1990 to 2010. *Blood* **123**(5), 615–624 (2014). 24297872 10.1182/blood-2013-06-508325 PMC3907750

[25] White, N. J. Anaemia and malaria. *Malar J.* **17**, 371 (2018). 30340592 10.1186/s12936-018-2509-9 PMC6194647

[26] Sawadogo, S., Niébié, K., Millogo, T. & Kafando, E. Blood transfusion requirements among children with severe malarial anemia: a cross-sectional study in a second level reference hospital in Burkina Faso. *Pan Afr. Med. J*. **37**(108) (2020). 10.11604/pamj.2020.37.108.22384 PMC7757275 33425141

[27] Alao, M. J., Joseph, A., Yakoubou, A., Agbodjogbé, Y., Gbénou, A. S. & Zoumenou, E. Urgences pédiatriques : Aspects épidémiologiques, cliniques, thérapeutiques et évolutifs au CHU de la Mère et de l’Enfant-Lagune (CHU-MEL) de Cotonou—Bénin en 20193 57. Société de l’Anesthésie Réanimation d’Afrique Francophone.; RAMUR Tome 25, n^o^2–2020. Disponible sur: https://www.calameo.com/read/00456829345c5983a1581

[28] Sène, M. Aspects épidémiologiques cliniques, paracliniques, évolutifs et thérapeutiques du paludisme grave de l’enfant au service de pédiatrie de l’Hôpital de la Paix de Ziguinchor. 2024 [cité 8 oct 2024]; Disponible sur: http://rivieresdusud.uasz.sn/xmlui/handle/123456789/2129

[29] World Health Organization(WHO). Worl malaria report 2022 [Internet]. Geneva; 2022 [cité 11 oct 2024]. 372 p. Disponible sur: https://www.who.int/teams/global-malaria-programme/reports/world-malaria-report-2022

[30] Hill, J., PDMC Saves Lives Consortium. Implementation of post-discharge malaria chemoprevention (PDMC) in Benin, Kenya, Malawi, and Uganda: Stakeholder engagement meeting report. *Malar J.* **23**(1), 89. 10.1186/s12936-023-04810-0 (2024). 38539181 10.1186/s12936-023-04810-0 PMC10976733

