# Supplementary material for: Prevalence and clinical impact of severe anaemia in referral hospitals in southern Benin
Source: Sci Rep. 2025 Jul 1;15:21431. doi: 10.1038/s41598-025-04298-5 (PMC12218909; doi:10.1038/s41598-025-04298-5)
Supplement: Supplementary file 1 — Supplementary Material 1 [file 41598_2025_4298_MOESM1_ESM.docx]

**Appendix**

**Appendix 1.** Breakdown by priority diagnosis of children hospitalised in paediatrics in 2023


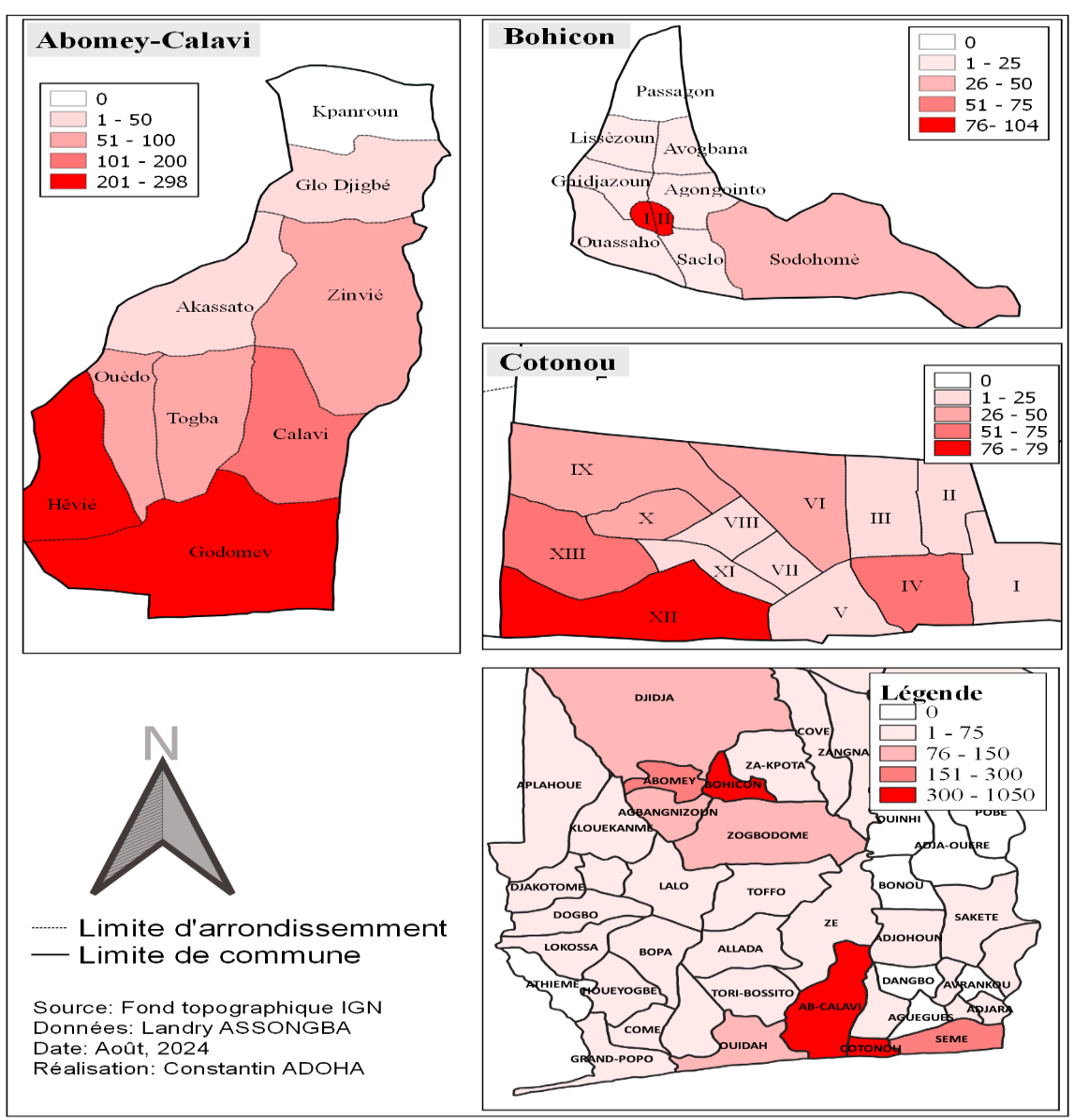

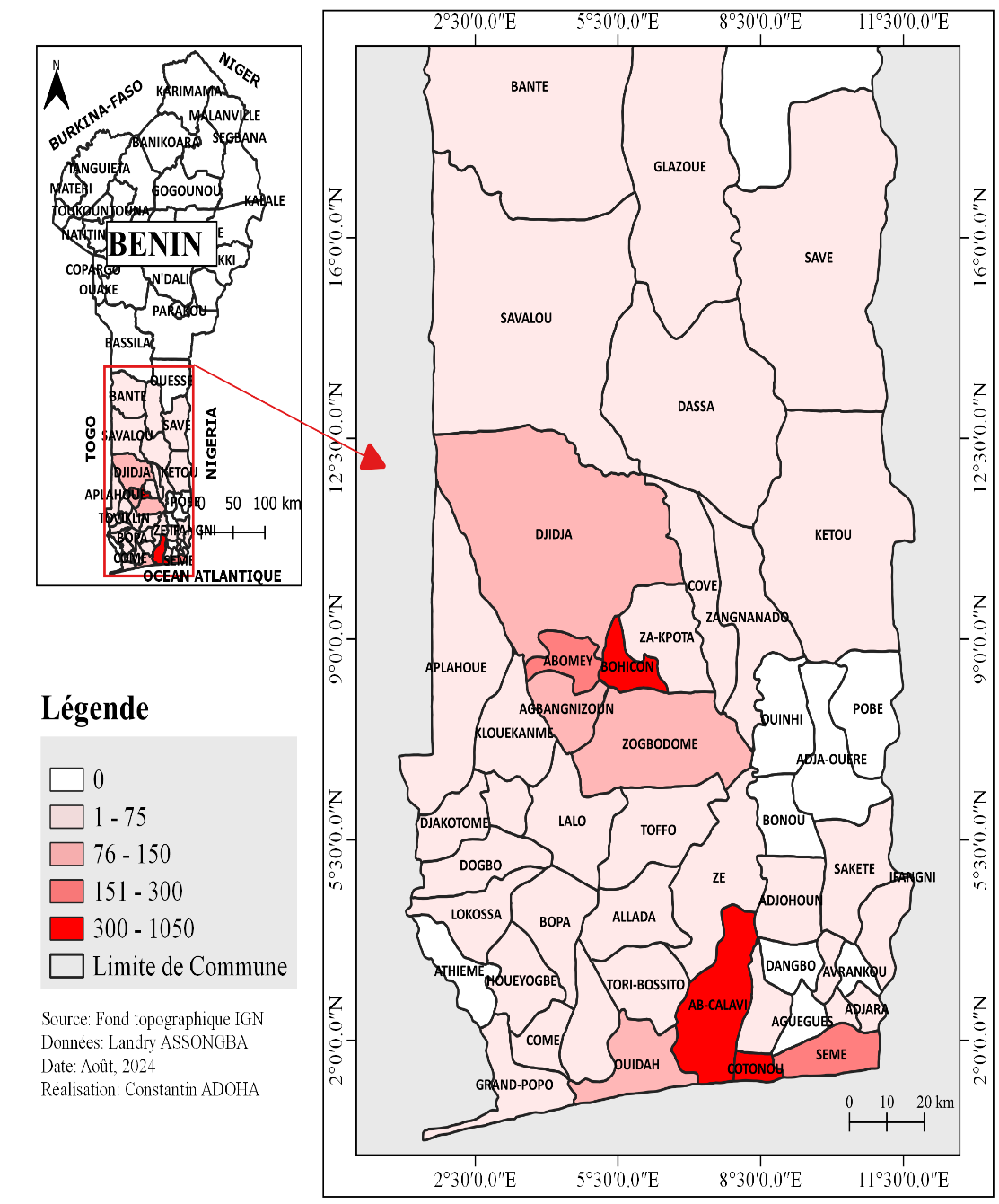
**Appendix 2**. Spatial variation in the geographic origin of severe anaemia

The authors generated the map using QGIS version 3.36.3 (<https://www.qgis.org>), an open-source geographic information system. The number of severe anaemia cases was plotted using coordinates corresponding to the districts of origin, initially aggregated at the district level. The map was then zoomed in on districts reporting more than 300 cases per year to show at the distribution at the sub-district level.
